# Electroacupuncture Reduces Heart Rate and Perceived Exertion During a Bike Test: A Preliminary Analysis

**DOI:** 10.3390/ijerph21101369

**Published:** 2024-10-17

**Authors:** Emily Gaudet, Tristan Castonguay, Maryse Fortin, Geoffrey Dover

**Affiliations:** 1 Department of Health, Kinesiology and Applied Physiology, Concordia University, Montreal, QC H4B 1R6, Canada; emilygaudet97@hotmail.com (E.G.); maryse.fortin@concordia.ca (M.F.); geoffrey.dover@concordia.ca (G.D.); 2 CRIR—Centre de Réadaptation Constance-Lethbridge, Montreal, QC H4B 1T3, Canada

**Keywords:** electroacupuncture, physical activity, weight loss, cardiovascular benefits, aerobic fitness, exercise recovery, blood pressure

## Abstract

Background: Preliminary research suggests that acupuncture can improve cardiovascular function. The purpose of our study was to determine if electroacupuncture can improve performance and post-exercise recovery. Methods: Thirty-two healthy people participated in this study (14 men and 18 women, aged 23.6 ± 3.5 years). The first visit included baseline measurements. Then, the participants received daily electroacupuncture at acupuncture point PC6 for a week, followed by a second visit. Heart rate, perceived exertion, and systolic and diastolic blood pressure were measured before, during, and after a YMCA submaximal bike test. Results: The heart rate was significantly reduced during the final stage of the YMCA test (151.3 ± 7.0 to 146.7 ± 11.8; *p* = 0.013) on the second visit. The rate of perceived exertion was significantly lower during all stages in Visit 2 (average RPE Visit 2 = 10.71 ± 2.02; average RPE Visit 1 = 11.45 ± 1.98; *p* = 0.004). Systolic blood pressure significantly decreased during the 5 min post-test recovery (SBP Visit 2 = 116.9 ± 12.0; SBP Visit 1 = 145.7 ± 14.6, *p* < 0.05). Conclusions: A week of electroacupuncture at PC6 led to reduced heart rate and perceived exertion during exercise, making the workload feel less strenuous. Electroacupuncture at PC6 shows potential for increasing participation in physical activities by making them feel easier to accomplish.

## 1. Introduction

Physical activity constitutes a vital component of lifestyle intervention that is integral to both weight loss and weight management programs [1]. Yet the merits of exercise encompass a broader goal than losing weight. Substantial evidence indicates that exercise reduces cardiovascular diseases by fostering a healthy, anti-inflammatory environment, and diminishing various risk factors associated with cardiovascular pathological conditions [2,3]. But unfortunately, it is estimated that only 54% of people who intend to be physically active actually achieve their goal [4]. Low-intensity workouts offer health benefits, but opting for higher intensity and longer duration exercises not only reduces the risk of cardiovascular diseases but also facilitates weight loss for the individual [5]. Overcoming barriers to physical activity for weight loss is essential due to the multifaceted challenges individuals often encounter [6]. In a 2019 article featured in the American Journal of Health Behavior, the most widely supported obstacle to engaging in physical activity was identified as the fatigue induced by physical activities [7]. Placing a stronger focus on initiatives that promote easily attainable exercise like walking and low- to moderate-intensity activities can help individuals overcome any difficulties they may have with participating in physical activity [8,9,10]. Along these lines, there is increasing evidence that acupuncture could improve both exercise performance and post-exercise recovery.

Acupuncture is a form of traditional Chinese medicine and has been used to treat various acute and chronic conditions including obesity and heart disease [11]. Acupuncture includes traditional needling as well as electroacupuncture. A previous study showed that using traditional acupuncture therapy on various points in the ear, alongside encouraging participants to engage in walking and maintaining dietary control, can facilitate weight loss [12]. Acupuncture’s effects on obesity appear to involve the concurrent modulation of the neuromodulator, immune, and endocrine systems [12,13]. In addition, previous studies indicate that acupuncture can have therapeutic effects on various types of hypertension and coronary artery diseases, specifically angina pectoris [14,15,16,17,18]. Based on the traditional Chinese medicine theory, angina pectoris commonly affects the heart and pericardium meridian, so the points located along this meridian, such as Neiguan (pericardium 6; PC 6), play an essential role in the treatment of heart and chest diseases [16]. On-going acupuncture treatment in angina pectoris patients has been shown to significantly reduce the number of angina attacks per week as well as significantly increase cardiac work capacity and exercise tolerance before experiencing chest pains [14,17]. Moreover, administering acupuncture on PC 6 can significantly reduce the intensity of the pain felt during exercise in angina patients [17]. Stimulation of PC 6 has been shown to improve cardiac function, enhance myocardial contractility, and increase coronary artery blood flow and myocardial oxygen supply in order to relieve angina pectoris [16,19,20]. The evidence is striking: for each extra 15 min of daily exercise falling within the 15 to 100 min range, there is an impressive 4% reduction in the risk of all-cause mortality [21].

The suggested cardiovascular benefits of acupuncture treatments have led to the idea that traditional acupuncture may also enhance exercise performance, post-exercise recovery, and weight loss. As such, acupuncture has been used on both the general population and athletes for a variety of conditions and with the intention of improving their exercise performance [22,23]. For example, a 5-week acupuncture stimulation intervention on anaerobic threshold and exercise capacity in healthy untrained males found a higher maximal exercise capacity before the onset of blood lactate, as well as lower heart rates at both submaximal and maximal exercise levels compared to a placebo group [24]. Improving exercise capacity is significantly beneficial for obese individuals and those who wish to experience weight loss.

Exercising at excessively high intensity levels can discourage sedentary individuals from engaging in physical activity, as it may induce negative emotions and potentially deter them from future exercise participation [25]. If acupuncture can reduce exercise-related challenges or enhance post-exercise recovery, it may assist sedentary individuals in overcoming obstacles to physical activity. Exercise recovery can be defined in a few ways. Recovery entails the process of re-establishing a state of homeostasis, akin to restoring resting cardiovascular parameters such as heart rate and blood pressure [26]. This intricate process entails the harmonized response of numerous systems, orchestrating the return of the body to a state of equilibrium [26]. The trajectory of cardiac autonomic recovery over time mirrors the re-establishment of cardiovascular balance, an integral facet of overall recovery [26]. The typical method for assessing post-exercise recovery involves tracking the return of resting cardiovascular parameters. This is typically accomplished by measuring heart rate recovery using the heart rate data collected during the initial 60–300 s after exercise [26]. Another way to monitor the cardiovascular stressor of exercise is to track the variation in blood pressure. Li et al.’s (2004) findings emphasize that acupuncture applied at specific acupoints (P 5–6 and LI 4-L7) may result in an improved workload during exercise even if the blood pressure is reduced [27]. In the context of our study, all our measurements post-exercise are referred to as post-exercise recovery.

Cardiac autonomic recovery is expedited in individuals with higher aerobic fitness, as evidenced by the study conducted by Stanley et al. in 2013 [26]. Therefore, our study aimed to assess the impact of electroacupuncture on parameters such as heart rate, perceived exertion, and blood pressure during exercise. We conducted these measurements both during the submaximal YMCA bike test and the subsequent post-exercise recovery phase. This investigation is our proof-of-concept that holds the potential to enhance the exercise experience and potentially overcome obstacles to engaging in physical activity that will lead to further testing in an obese population.

## 2. Materials and Methods

We performed a sample size calculation to assess the changes in heart rate, exertion, and blood pressure from baseline to the final stage of a maximal cycle ergometer test. According to Urroz et al. (2016), the effect size for heart rate following a maximal cycle ergometer test combined with acupuncture was found to be 1.8 [28]. However, due to differences in our study’s design, we chose a more conservative effect size of 0.5. Using SPSS 29 (IBM, Armonk, NY, USA) for paired means, we determined that a minimum of 30 participants was necessary to detect an effect size of 0.5 with 80% power and a significance level of 0.05. A sample of 32 healthy adult participants (14 men and 18 women) with a mean age of 23.6 ± 3.5 years of age (mean ± SD) were recruited from the university population. For participants to be included, participants needed to participate in 150 min of moderate to vigorous intensity aerobic exercise per week and have a body mass index score between 18.5 and 24.9 kg/m^2^. The participants were excluded if they smoked; had cardiovascular and neuropathic conditions, diseases, or disorders, or elevated blood pressure; were taking medication that can impact cardiovascular function; were suffering from acute inflammation; had a hemorrhagic tendency, arrhythmias, or epilepsy; had a metal implant or cardiac pacemaker; had active lower body injuries preventing cycling; or had a history of acupuncture use. We designed this study around a group of healthy participants in our prospective study to minimize the variability introduced by pre-existing health conditions. We recruited a homogeneous healthy group to reduce other health-related factors that could have impacted perceived exertion and heart rate. Additionally, healthy participants are less likely to experience complications from the intervention.

Written informed consent was received from all subjects after they were given the time to fully understand the objectives, methods, and potential risks associated with this study. All protocols were reviewed and approved by The Ethics Committee of Concordia University (Certificate: #30015225).

### 2.1. Equipment

#### 2.1.1. Electroacupuncture—ObeEnd Device

**Figure 1:**
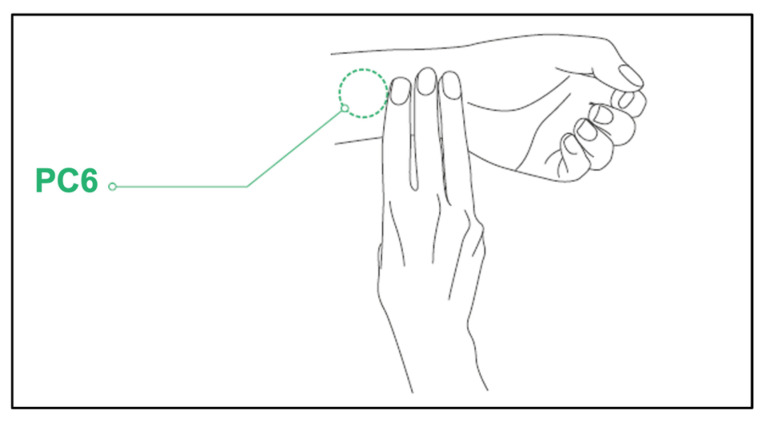
The location of the PC6 acupuncture point is 4–5 cm above the wrist line.

The ObeEnd device is a wearable device that uses transcutaneous electrical nerve stimulation (TENS) to continuously send targeted electrical pulses to acupuncture point PC 6 (i.e., Neiguan or pericardium 6). At one end of the device is an embedded TENS unit that is placed 4 to 5 cm from the transverse wrist lines, with a width of about three fingers to stimulate the PC 6 acupuncture point (see Figure 1). We showed the participants how to operate the band and all participants were instructed to increase the intensity of the electrostimulation until a comfortable tingling sensation was felt. The band was pre-programmed to apply the stimulation for 30 min upon being turned on.

#### 2.1.2. EDANiM50 Patient Monitor

The EANDiM50 (EDAN Instruments, Inc., Shenzhen, China) is a patient monitor used to record various physiological markers. For our study, we used the EDANiM50 3-lead echocardiogram to continuously monitor heart rate (HR). The EDANiM50 sphygmomanometer was used to measure blood pressure (BP). Both the systolic (SBP) and diastolic blood pressure (DBP) values were recorded. Taking the blood pressure measurements requires pressing a button on the monitor to inflate the cuff, and the measurement takes approximately 30–40 s. The baseline blood pressure readings should be taken at rest since activity can alter the blood pressure reading [29].

### 2.2. Measures

#### 2.2.1. Rate of Perceived Exertion (RPE)

RPE is a valuable measure of workload and has been validated against heart rate with excellent results (r = 0.80–0.90) [30]. RPE has been used in physiotherapy and sports science research extensively because the RPE score increases linearly with exercise intensity, and is, therefore, a valuable measure when researchers want to quantify responses to exercise [31,32]. The Borg Scale of Perceived Exertion is a quantitative measure that is widely used to assess subjective perception of an individual’s effort during exercise and is a highly relevant tool for occupational health and safety practices [33]. The American College of Sports Medicine (ACSM) recognizes that RPE can add precision to heart rate recordings when monitoring exercise intensity [34]. The Borg scale scores range from 6, indicating no exertion at all, to 20, indicating maximal exertion. We used the Borg scale to measure the perceived exertion during the bike test [34].

#### 2.2.2. Incremental Exercise Testing—YMCA Test

Submaximal cycling tests are often performed for entry to cardiac and pulmonary rehabilitation as well as for establishing the safety of exercise participation for adult fitness and wellness programs [35]. The YMCA submaximal test consists of multiple stages of increasing load performed on a Monark Ergomedic 828 E Cycle Ergometer (Monark, Sweden). The YMCA test was chosen because it has a good association with RPE and has been used in published research before [35]. During the test, only the “revolutions per minute” (RPM) counter was kept visible on the bike screen to allow the participant to maintain a cadence of 50 rpm. Keeping a consistent cadence is crucial for sustaining the required level of effort and preventing undesirable effects. Pedalling at a high cadence (>95 RPM) can impact the overall exertion, while a low cadence (<40 RPM) can intensify leg muscle and knee pain, ultimately changing the overall perceived exertion [36]. Heart rate was recorded at the end of every minute of the submaximal test. At the end of each stage, the blood pressure and RPE were recorded.

The submaximal test began with a 3 min warm-up with no resistance. The load was then increased to 0.5 kilopond (kp) to start the first stage of the test. The initial workload for the test was established based on the heart rate recorded at the end of the first stage. Each stage of the test lasted 3 min. If the participant was unable to maintain a steady-state heart rate within the last two minutes of a stage (+/−5 bpm), another minute was added to that stage until a steady-state heart rate was achieved. If the heart rate was lower, the power output for the subsequent stages was higher, and conversely, a higher heart rate resulted in a lower power output. Each subsequent stage was increased by a load of 0.5 kp. Heart rate, blood pressure, and RPE were all required measurements in the YMCA protocol to ensure the selection of a proper starting load and to monitor the participant’s effort and safety throughout the test. The aim of the test was to achieve two consecutive workloads where the heart rate was between 110 bpm and 85% of the age-predicted HRmax (220-age).

### 2.3. Protocol

All participants attended two laboratory visits that were scheduled 7 days apart. The duration of a week is common in acupuncture research as other studies have used a short stimulation experiment (from 1 day to 1 week) to measure cardiovascular changes post-acupuncture. [15,22,37,38,39,40,41,42,43] all studied the effects of a single acupuncture intervention and the effect on the cardiovascular system. The results were mostly not significant and suggested a longer intervention period. Other short-term research projects studied the effect of acupuncture for a week and found beneficial results from acupuncture on the cardiovascular system. For instance, Park et al. (2010) discovered a notable enhancement in flow-mediated dilation of the brachial artery with acupuncture at acupoint ST 36, as well as with the combination of ST 36 and PC6 [42]. Consequently, we structured our experiment to include daily stimulation over a week, as we anticipate observing effects on the cardiovascular system during that timeframe.

**Figure 2:**
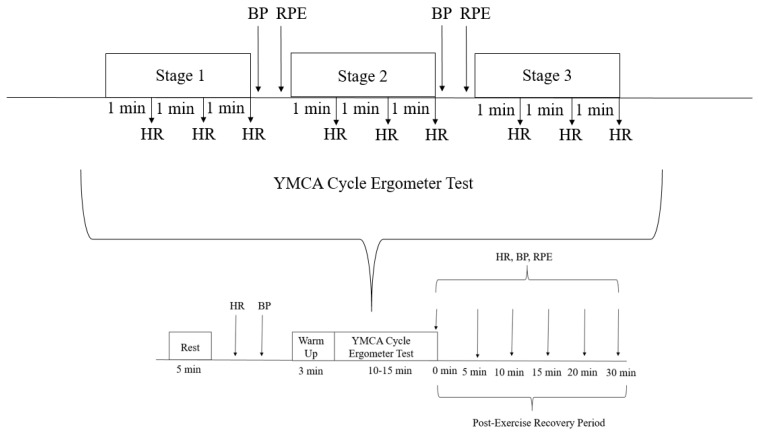
Outline of the participant’s session.

Prior to each visit, participants were instructed to refrain from exercising and consuming alcohol, nicotine, cannabis, and caffeine for at least 12 h. Please refer to Figure 2 for the outline of this study’s timeline.

For the first visit, a script was read to participants to ensure consistency of the information provided to all participants. All participants filled out a demographic questionnaire, the International Physical Activity Questionnaire, and the CSEP-Get Active Questionnaire for exercise clearance purposes. Information from the demographic questionnaire was used to calculate body mass index, 85% MHR, and estimate maximal oxygen consumption (VO2max).

Baseline: The participants were seated and linked to the EDANiM50 patient monitor. The participants remained seated for 5 min while they were given clear instructions on the YMCA protocol and how to use the Borg scale. Baseline heart rate and blood pressure were recorded during the 5 min explanation period. The participants were then brought to the cycle ergometer, where their seat height was adjusted and recorded for the second visit.

VO2 testing: The YMCA submaximal exercise test began with a warm-up period followed by the stages of the exercise test. Heart rate was recorded at every minute of every stage.

Post-exercise recovery: At the end of the test, the participants were instructed to get off the bike and were then brought back to a chair to start the post-exercise recovery period. The last blood pressure reading during the exercise test was used as the first blood pressure reading for the post-exercise recovery period. Heart rate and RPE for their “current level of perceived exertion” were recorded as soon as the participant sat down, yielding the measurements for the “0 min” mark. Heart rate, blood pressure, and RPE measurements were continuously recorded for up to 30 min or until values returned to baseline measurements.

At the end of Visit 1, participants were given an instruction manual with clear verbal instructions on how to use the electrostimulation feature on the ObeEnd band. The participants were instructed to use the stimulation 3 sessions a day, daily, for 30 min each session until the next visit approximately one week later. This treatment provides a total of 90 min of daily stimulation of the PC6 acupuncture point.

On the second visit, participants were instructed to carry out one last round of stimulation (lasting approximately 15 min) at the beginning of their session. Throughout this period, the participants were interviewed to gather follow-up information about their present condition and their experience with the band during the previous week. Subsequently, the identical procedure utilized during the initial visit including the submaximal bike test and measurement of HR, blood pressure, and perceived exertion were measured.

### 2.4. Data Analysis

#### 2.4.1. Visit 1 Analysis of RPE and Cardiovascular Variables

We used SPSS 29.0.2.0 (IBM, USA) for all our statistical testing. We checked the normality and heterogeneity of all the data. In case we found a statistically significant interaction, we conducted follow-up *t*-tests for each measure to identify which one showed significant differences.

The first analysis aimed to evaluate the changes in heart rate and blood pressure during the YMCA test. We conducted this analysis independently for each participant, considering data from before, during, and after the test (Visit 1).

Since each participant completed a different number of stages, we focused our analysis on stage 1 (the easiest stage) and the last stage (the hardest), as these stages were completed by all participants. We used a one-way ANOVA to compare the heart rate, systolic blood pressure, and diastolic blood pressure at baseline, stage 1, stage 2, and the last stage for all participants.

Additionally, we examined the changes in perceived exertion using a one-way ANOVA, but we only had data for RPE during stage 1, stage 2, and the last stage, as RPE was not recorded during the baseline.

#### 2.4.2. Comparison of Visit 1 and Visit 2

We conducted two main analyses to assess the effects of using the ObeEnd band for a week. Firstly, we employed a 2 × 4 repeated measures ANOVA to investigate changes in heart rate, systolic blood pressure, and diastolic blood pressure between Visit 1 and Visit 2.

Secondly, we conducted a 2 × 3 repeated measures ANOVA to examine changes in perceived exertion between Visit 1 and Visit 2. Prior to these analyses, we checked for normality and heterogeneity of all data. In cases where sphericity was violated, we applied the Greenhouse–Geisser correction.

In the event of finding a statistically significant interaction, we proceeded with a *t*-test for each measure to identify which specific changes were significantly different between Visit 1 and Visit 2.

#### 2.4.3. Comparison of Post-Exercise Recovery Periods

As part of our experiment, we investigated the cardiovascular variables during the post-exercise recovery period after each YMCA test. To achieve this, we used a paired *t*-test to compare the measurements taken before and after the test. We focused on the following cardiovascular variables: heart rate, systolic blood pressure, diastolic blood pressure, and the rate of perceived exertion for both Visit 1 and Visit 2. However, we excluded the post-exercise recovery after 5 min from our analysis, as not all participants experienced equal recovery times.

## 3. Results

Table 1 presents the values gathered during Visits 1 and 2 at various stages of the YMCA submaximal exercise test, while Table 2 details the recovery data.

### 3.1. Visit 1 Analysis of Cardiovascular Variables and RPE

Heart rate: The one-way ANOVA indicated a significant difference in heart rate between stages on Visit 1 (F = 266.322, *p* < 0.001). The Tukey Post Hoc analysis indicated that there was a significant increase in heart rate between baseline and stage 1 (95% CI: −33.9–−18.1, *p* < 0.001), stage 2 (95% CI: −60.8–−44.9, *p* < 0.001), and the last stage (95% CI: −89.6–−73.7, *p* < 0.001) of the submaximal VO2 test.

Systolic blood pressure: The one-way ANOVA indicated a significant difference in SBP during stage 1 on Visit 1 (*p* < 0.001). The Tukey Post Hoc analysis revealed a significant increase in SBP between baseline and stage 2 (95% CI: −30.9–−9.8, *p* < 0.001), baseline and the last stage (95% CI: −42.2–−21.0, *p* < 0.001), stage 1 and stage 2 (*p* = 0.002), and between stage 1 and the last stage (F = 81.595, *p* < 0.001).

Diastolic blood pressure: The one-way ANOVA indicated no significant increase in DBP between Visit 1 baseline, stage 1, stage 2, and the last stage of the test (*p* = 0.069).

Rate of perceived exertion: The one-way ANOVA revealed a significant increase in RPE for each stage of the submaximal bike test (F= 173.367, Greenhouse–Geisser correction for *p* < 0.001). The Tukey Post Hoc analysis indicated a statistically significant increase in RPE between stage 1 and stage 2 (95% CI: −4.385–−2.021, *p* < 0.001) and the last stage (95% CI: −8.229–−5.865, *p* < 0.001).

### 3.2. Comparison of Visit 1 and Visit 2

Heart rate: The 2 × 4 repeated measures ANOVA revealed a significant interaction between visit and stage for heart rate (F = 6.035 Greenhouse–Geisser correction for *p* = 0.004). A separate dependent *t*-test indicated a significant decrease in heart rate during the last stage on Visit 2 compared to Visit 1 (t = 2.627, *p* = 0.013).

Blood pressure: The other 2 × 4 repeated measures ANOVA indicated no significant interaction between visit and stage for SBP or DBP (F = 0.17, *p* = 0.916 and F = 0.478, *p* = 0.699, respectively).

Rate of perceived exertion: The 2 × 3 repeated measures ANOVA indicated no significant interaction between visit and stage for RPE (F = 0.959, *p* = 0.389). However, it is worth noting that a significant main effect (F = 10.745, *p* = 0.003) indicated a significant difference in RPE during Visit 2 versus Visit 1. The average RPE on Visit 1 was 11.45 ± 1.98, which was reduced to 10.71 ± 2.02 during Visit 2.

### 3.3. Comparison of Post-Exercise Recovery Periods

While the values were decreased during the post-exercise recovery phase, there was no statistically significant change in average heart rate and average diastolic blood pressure when comparing Visit 1 and Visit 2 with a dependent *t*-test.

At the “5 min” mark of the post-exercise recovery period, there was a statistically significant decrease in systolic blood pressure on Visit 2 (116.9 ± 12.0) compared to Visit 1 (120.1 ± 11.3) (*p* = 0.022, see Table 2).

## 4. Discussion

Most of the research on acupuncture has been on traditional acupuncture and health, but there is some evidence that electroacupuncture may be effective as well [27,28,44,45]. There is limited research on the cardiovascular effects of electroacupuncture on exercise performance and post-exercise recovery. Therefore, our study aimed to investigate the effect of electroacupuncture on cardiovascular variables during a submaximal cardiovascular test as well as the recovery period afterward. There is some previous evidence that supports the use of electroacupuncture stimulation of PC 6 to enhance exercise performance and post-exercise recovery from submaximal exercise [28,44].

The key findings in our study were that after one week of PC6 electroacupuncture stimulation, the average heart rate was statistically significantly lower in the last stage of the submaximal exercise test and the systolic blood pressure was lower 5 min into the post-exercise recovery period. Another intriguing discovery was the significant main effect observed for the rate of perceived exertion. On average, RPE during the submaximal exercise test in Visit 2 was found to be significantly lower compared to Visit 1. This means that after wearing the band for one week, participants were able to complete the hardest workload of the submaximal VO2 test with a lower heart rate, recover quicker after the same workload, and perceive less exertion on average. While the last stage RPE on the second visit continued to decrease compared to Session 1, this difference was very close but not statistically significant (*p* = 0.051), and only the main effect was statistically significant. This finding is very important as it links fatigue to exercise adherence [46]. Increased intensity in physical activity has been linked to more significant enhancements in health and fitness [47]. However, this may come at the expense of potentially lower levels of adherence [48]. There is evidence that people do not exercise because it is too hard and tiring [46,49]. A meta-analysis from 2020 reported that elevating exercise intensity was found to have a great negative impact on adherence [50]. Burnet et al. (2020) note that increasing exercise intensity can significantly negatively impact adherence to exercise [50]. Therefore, finding a way to affect those barriers to exercise by decreasing the exercise difficulty might help with exercise participation and adherence. Our analysis suggests that electroacupuncture can diminish the perceived intensity and difficulty of exercise. As a result, we speculate that lowering perceived exertion might reduce barriers for some individuals, encouraging them to participate in more physical activity.

During the first visit, the statistically significant increase in heart rate, systolic blood pressure, and rate of perceived exertion for each stage of the YMCA protocol indicates that this was an appropriate submaximal test for this study. As the intensity of exercise increases, there are corresponding and interdependent changes in both perceptual and physiological responses [51]. Thus, the steady and significant increase in heart rate and systolic blood pressure throughout the test reflected the increase in the participant’s perceived exertion with increasing loads. This test also provided enough increase in difficulty in each variable to allow us to see the change in RPE after using the band. A score of 17 would be representative of a healthy person being able to continue but having to push themselves beyond their feeling of being very fatigued [33]. The final stage of the exercise test chosen reached the level of “Hard” on the Borg scale [34,52] in both Session 1 (15.08 ± 1.98) and Session 2 (14.56 ± 2.37), demonstrating an appropriate difficulty for the submaximal nature of the test.

Previous research has indicated that there is a high correlation between a person’s perceived exertion rating and their actual heart rate during exercise. A Borg RPE scale of 6 corresponds to a heart rate of 60 beats/min in a healthy adult [52]. This further demonstrates the accuracy of our test since our participants were able to maintain the correlation between RPE and heart rate at the last stage of the submaximal exercise test in both Session 1 (151.3 ± 7.0) and Session 2 (146.7 ± 11.8).

In cases of obesity, functional limitations are prevalent, affecting nearly 40% to 70% of adults [53]. A sedentary lifestyle, coupled with insufficient engagement in regular physical activity, leads to detrimental consequences [54]. The significance of physical activity in fostering health cannot be understated. Evidence suggests a linear relationship between the quantity of physical activity and its benefits [1]. It is in the context of decreasing mortality risks that the potential of electroacupuncture emerges as a transformative strategy. By facilitating intensified exercise performance and hastened post-exercise recovery, electroacupuncture could galvanize individuals to embrace physical activity with renewed enthusiasm. In doing so, it may dismantle some of the functional barriers that have hindered widespread participation, opening doors to a healthier and more active population.

We postulate two theories based on published evidence on the reasons why electroacupuncture may be affecting the cardiovascular system during exercise. The first theory is from Nishijo et al. 1997 [55]. The researchers analyzed the cardiac effects of acupuncture stimulation of P4 in healthy subjects in conjunction with dosages of atropine (a muscarinic receptor antagonist) and propranolol (a β-adrenergic receptor antagonist) and found a typical bradycardia response, with the decrease being about 10% of the basal heart rate [55]. They concluded that this decrease in heart rate following acupuncture was a result of a reflex reciprocal coordination of an increase in cardiac vagal activity and a decrease in cardiac sympathetic activity [55]. The research from Zhou and Longhurst in 2012 contributed more information about the decrease in sympathetic activity. It is suggested that acupuncture can affect the neuroendocrine mechanisms through the stimulation of neurons in the arcuate nucleus, ventrolateral gray matter, and raphe nuclei [56]. The stimulation of the neurons helps inhibit angiotensin type 1 (AT1) receptors in the rostral ventrolateral medulla [56]. This action decreases sympathetic nerve activity and lowers blood pressure [56]. Although our study analyzed exercise-induced heart rate as opposed to resting heart rate, it is interesting to note that the significant change in heart rate seen in the last stage of the submaximal exercise test was 9.6% lower in Session 2 (146.7 ± 11.8) compared to Session 1 (151.3 ± 7.0). Further research is needed to investigate this correlation between acupuncture stimulations and the bradycardic response during exercise, which could be due to the reciprocal reflex that affects the vagal activity or the suppression of the sympathetic nervous system.

The second theory is based on the work of multiple researchers who looked into pain inhibition mechanisms of acupuncture [57,58,59,60]. The ability to perform the same workload at a lower perceived exertion could possibly also be attributed to acupuncture’s effects on pain inhibition. Currently, some studies of the mechanism of action of acupuncture and electroacupuncture stimulation show the essential role of the release of endogenous opioid peptides into the central nervous system (CNS) [57,58,59,60]. These β-endorphins can aid in pain modulation and inhibition of nociceptive transmission at all levels of the nervous system, resulting in long-lasting pain control [23]. Since increased concentrations of β-endorphins have been found following both exercise and the application of acupuncture, acupuncture has been hypothesized to attenuate the sympathetic nervous system (SNS) at the level of the CNS [23]. While our participants were not in pain, they did achieve a score of 15 on the Borg scale, which is representative of an exercise-induced stressor imposed on the body as a result of the submaximal exercise test [52]. Thus, the above-mentioned analgesic mechanism initiated by one week of acupuncture stimulations could explain the small but statistically significant decrease in RPE when performing the same workload one week after wearing the band.

Additionally, Dhillon (2008) [45] examined the effects of acupuncture on semi-competitive and competitive road or mountain cyclists and showed statistically and clinically significant increases in RPE scores in the acupuncture group (17.65 ± 0.67) compared to the sham group (16.95 ± 0.99) and the control group (16.85 ± 0.88) at the 20 km mark (*p* = 0.0088) [45]. This was accompanied by the acupuncture groups clinically significant reduction in time to completion of the 20 km (36.19 ± 5.23) in comparison to the sham (37.03 ± 5.66) and control groups (37.48 ± 6.00) (*p* = 0.76), indicating that the acupuncture group was able to significantly increase their ability to exert themselves by cycling faster than the others [45]. Dhillon’s study showed improvements in exercise performance after the use of acupuncture through a higher peak RPE, resulting in a higher workload intensity and a faster time to completion [45]. Whereas our study showed improvements in exercise performance and recovery after the use of acupuncture through a lower RPE throughout the exercise test conducted over a fixed time and workload intensity.

Post-exercise recovery can be defined in a few ways. One interpretation is having a reduced heart rate after completion of the exercise, indicating that the exercise was not as challenging, and the person recovered faster. Another interpretation could be that exercise recovery is the ability of the participant to improve their physiological responses to the same workload of exercise. This is because we are not assessing improvements in performance, but rather the ability to perform the same workload with more ease. As a result, we believe that reducing perceived exertion may help lower barriers for some individuals, encouraging greater participation in physical activity.

### Study Limitations

The participants in this study only received the acupuncture treatments over the course of one week. Acupuncture and other rehabilitation interventions are usually longer than one week, which would limit the effectiveness of our intervention. However, there are examples of a one-session application of acupuncture being used in previous studies. A study conducted by Karvelas et al. (1996) [22] analyzed the effects of single-use acupuncture treatment prior to both maximal and submaximal graded exercise tests and concluded no significant immediate effect on perceived exertion and physiological responses [22]. However, other references like a 2021 article from Kimura et al. [37], observed a significant decrease in blood pressure and heart rate after a single acupuncture treatment [37,38]. More studies are needed to measure the efficacy of longer acupuncture treatments on perceived exertion and heart rate.

Additionally, the response to acupuncture is variable as clinical experience suggests that approximately 20% to 50% of the population is nonresponsive to acupuncture in the treatment of both acute and chronic painful conditions [61].

Since the participants used the band on their own time, subjective perception of stimulation guided their intensity setting, where some participants had higher pain tolerances compared to others, therefore, using higher intensity of stimulation. Those who utilized lower stimulation intensities might not have activated the central opioid system, and as a result, could not benefit from the mechanism by which acupuncture is thought to function through.

Relying on a subjective measure of exercise intensity may be considered a limitation of this study. However, we chose to utilize the Borg scale due to its support in the literature for assessing exercise intensity. To address potential concerns about subjectivity, we complemented these subjective measures with rigorous statistical analyses that correlated the reported intensity with objective data from heart rate and blood pressure.

## 5. Conclusions

This study served as the proof-of-concept to investigate whether electroacupuncture could facilitate exercise in a healthy population. The conceptualization and research design of this study focussed on individual differences. One week of electrical stimulation of acupuncture point PC6 leads to a significantly lower RPE and heart rate during the last stage of a submaximal test. In addition, after the 5 min post-YMCA submaximal test, there was a significant decrease in systolic blood pressure (SBP) during the post-exercise recovery (SBP Visit 1 = 145.7 ± 14.6, SBP Visit 2 = 116.9 ± 12.0, *p* < 0.05) when comparing the values of both visits. This finding indicates a statistically significant reduction in systolic blood pressure during the recovery period following the submaximal exercise test. Thus, the electroacupuncture stimulation of PC 6 allowed the participants to exercise at the same workload with less physical and less perceived effort, showing preliminary evidence of the ObeEnd band’s ability to enhance exercise recovery. Simplifying the ability to engage in higher-intensity exercise and expediting post-exercise recovery could effectively encourage individuals to participate in more physical activities. As a result, they could reap the rewards of improved health outcomes. Based on our findings, the next logical step would be to conduct a randomized clinical trial using a clinically relevant population as well as a placebo band to further validate our results and the efficacy of electroacupuncture in decreasing the difficulty of physical activity.

## Figures and Tables

**Table 1:** Visits 1 and 2 mean SBP, DBP, and HR at different stages of the YMCA submaximal exercise test. ^a^ Indicates a significant difference (*p* < 0.01) between the SBP values of different stages. ^b^ Indicates no significant difference but a large increase in SBP. ^c^ Indicates a significant difference (*p* < 0.05) between HR on Visit 1 compared to Visit 2. ^d^ Indicates a significant main effect between RPE scores between the two visits (*p* = 0.003).

Visit 1	Baseline	Stage 1	Stage 2	Last Stage	Stage 1, 2, and Last Stage Average
HR (bpm ± SD)	69.6 ± 12.6	95.6 ± 14.3	122.5 ± 13.5	151.3 ± 7.0	
SBP (mmHg ± SD)	116.7 ± 13.4 ^a,b^	121.8 ± 15.5 ^a,b^	137.0 ± 17.9 ^a^	148.3 ± 17.7 ^a^	
DBP (mmHg ± SD)	67.1 ± 7.6	66.5 ± 12.3	68.4 ± 9.9	71.3 ± 9.3	
RPE score ± SD		8.03 ± 1.91	11.23 ± 2.07	15.08 ± 1.98	11.45 ± 1.98
Visit 2	Baseline	Stage 1	Stage 2	Last Stage	
HR (bpm ± SD)	71.8 ± 9.8	98.0 ± 15.7	120.8 ± 15.6	146.7 ± 11.8 ^c^	
SBP (mmHg ± SD)	114.8 ± 12.4	120.4 ± 16.3	134.0 ± 15.6	145.7 ± 14.6	
DBP (mmHg ± SD)	67.1 ± 7.6	66.5 ± 12.3	68.4 ± 9.9	71.3 ± 9.3	
RPE score ± SD		7.28 ± 1.43 ^c^	10.34 ± 2.26 ^c^	14.56 ± 2.37	10.71 ± 2.02 ^d^

**Table 2:** Post-exercise recovery cardiovascular variables at the 0 and 5 min mark after the YMCA test. ^c^ Indicates a significant difference (*p* < 0.05) between SBP on Visit 2 compared to Visit 1.

Post-Exercise Recovery Cardiovascular Variables	0 min After Test	5 min After Test
HR Visit 1 (bpm ± SD)	120.1 ± 11.0	85.4 ± 9.7
HR Visit 2 (bpm ± SD)	116.5 ± 14.0	83.1 ± 12.1
SBP Visit 1 (mmHg ± SD)	148.0 ± 17.7	120.1 ± 11.3
SBP Visit 2 (mmHg ± SD)	145.7 ± 14.6	116.9 ± 12.0 ^c^
DBP Visit 1 (mmHg ± SD)	73.1 ± 11.1	69.2 ± 10.2
DBP Visit 2 (mmHg ± SD)	71.3 ± 9.3	68.5 ± 8.5
RPE score Visit 1 ± SD	10.23 ± 2.95	7.03 ± 1.43
RPE score Visit 2 ± SD	10.44 ± 3.03	7.27 ± 1.82

## Data Availability

The data acquired for this study are available from the corresponding authors upon reasonable request.

## Correction Statement

Following the removal of this article from the Special Issue “The Effect of the Physical Activity on the Health of People”, an Editorial Board member reviewed the entire editorial process and confirmed all elements had been carried out as per MDPI Editorial Process policy (https://www.mdpi.com/editorial_process). As a result, the original Academic Editor listed on this publication has been replaced with that of the Editorial Board member who conducted this review. The original publication has now been updated accordingly. This information has also been transmitted to relevant indexing organizations.
